# An audit of the Beaumont Hospital Acute Stroke Unit and the effectiveness of the Irish FAST campaign

**DOI:** 10.1186/1753-6561-6-S4-O28

**Published:** 2012-07-09

**Authors:** D Zeng, L Mellon, A Hickey, D Williams

**Affiliations:** 1 Royal College of Surgeons in Ireland

## Introduction

Stroke is the second leading cause of death worldwide, and the leading cause of acquired disability in adults. An audit conducted by the Irish Heart Foundation revealed that there are inadequate services and facilities to prevent, assess and treat the yearly 10 000 stroke victims in Ireland. A national media campaign (FAST) was broadcast to educate people about the warning signs of stroke. This audit is to examine the effectiveness of the Beaumont Hospital Acute Stroke Unit and the FAST campaign.

## Methods

Information regarding stroke admissions to Beaumont Hospital was collected during the FAST campaign period of April, 2010 to June, 2011. The data was retrieved from the stroke-coordinator in Beaumont and also through the hospital patients' database. The data included clinical details of the admission such as presenting symptoms, length of stay, and administration of thrombolysis.

## Results

A total of 501 patients were admitted to the stroke service. The average age was 70.5 years old. 62.3% of patients had a stroke and 10% had TIA. 86.5% were ischemic stroke while 13.5% were hemorrhagic stroke. 69.3% of the patients were admitted to the Acute Stroke Unit followed by 20.7% being admitted to medical beds. The average length of stay in the stroke unit for stroke patients was 9.2 days and 4.5 days for TIA patients. 6.4% of the patients received thrombolysis. The biggest risk factor for stroke patients was hypertension (51.9%). The most common presenting symptoms of patients was speech disturbance 29.6%, The FAST message was able to identify 76.8% of stroke and TIA patients.

## Conclusions

There is some room for improvement for the Beaumont Hospital Acute Stroke Unit especially in the rate of thrombolysis and the number of days in which stroke patients stay in the stroke unit. The FAST campaign is adequate for identifying the majority of warning signs of stroke, however this audit was unable find any any increase in patients admitted with stroke-like symptoms during the study period.

